# Aggregate-State Effects in the Atomistic Modeling of Organic Materials for Electrochemical Energy Conversion and Storage Devices: A Perspective

**DOI:** 10.3390/molecules25092233

**Published:** 2020-05-09

**Authors:** Sergei Manzhos

**Affiliations:** Centre Énergie Matériaux Télécommunications, Institut National de la Recherche Scientifique, 1650 boulevard Lionel-Boulet, Varennes, QC J3X1S2, Canada; sergei.manzhos@emt.inrs.ca; Tel.: +1-514-228-6841

**Keywords:** organic battery, perovskite solar cell, organic solar cell, charge transport later, ab initio modeling, organic solid

## Abstract

Development of new functional materials for novel energy conversion and storage technologies is often assisted by ab initio modeling. Specifically, for organic materials, such as electron and hole transport materials for perovskite solar cells, LED (light emitting diodes) emitters for organic LEDs (OLEDs), and active electrode materials for organic batteries, such modeling is often done at the molecular level. Modeling of aggregate-state effects is onerous, as packing may not be known or large simulation cells may be required for amorphous materials. Yet aggregate-state effects are essential to estimate charge transport rates, and they may also have substantial effects on redox potentials (voltages) and optical properties. This paper summarizes recent studies by the author’s group of aggregation effects on the electronic properties of organic materials used in optoelectronic devices and in organic batteries. We show that in some cases it is possible to understand the mechanism and predict specific performance characteristics based on simple molecular models, while in other cases the inclusion of effects of aggregation is essential. For example, it is possible to understand the mechanism and predict the overall shape of the voltage-capacity curve for insertion-type organic battery materials, but not the absolute voltage. On the other hand, oligomeric models of *p*-type organic electrode materials can allow for relatively reliable estimates of voltages. Inclusion of aggregate state modeling is critically important for estimating charge transport rates in materials and interfaces used in optoelectronic devices or when intermolecular charge transfer bands are important. We highlight the use of the semi-empirical DFTB (density functional tight binding) method to simplify such calculations.

## 1. Introduction

The story of the development of novel energy conversion and storage technologies, including novel types of solar cells, such as organic solar cells (OSC) [1] and perovskite solar cells (PSC) [2], electrochemical batteries [3], organic [4] and perovskite [5] light-emitting diodes (OLED and PLED, respectively), etc., is largely a story of developing the related functional materials. Materials providing key functionalities are often organic semiconductors. Those include donors and acceptors of organic solar cells (OSC), emitters and hosts of OLED, active electrode materials of organic batteries [6], and electron and hole transport layers in OSC, PSC, and PLED [7]. The advantages of organic materials include solution processability, and therefore amenability to large scale production; tunability of properties by choice of functional groups; and often, ease of synthesis or low cost. Both molecular and polymeric organic materials are used in these applications. Importantly, in a device, organic materials are used as thin films or blends and it is molecular packing that determines or modifies key electronic properties, including optical properties and electronic and ionic conductance. 

Development and characterization of organic functional materials is often assisted by ab initio modeling, typically at the density functional theory (DFT) [8,9] level. For materials considered for the technologies mentioned above, such modeling is most often done at the single-molecule level for molecular materials or oligomers for polymers. Estimates of HOMO (highest occupied molecular orbital) and LUMO (lowest unoccupied molecular orbital) levels, the bandgap, and absorption and emission spectra can be quite reliable from such molecular models. The simplicity of the computational setup, availability of easy-to-use codes, such as Gaussian [10], and speed of calculation favor such models. High-level DFT calculations with hybrid functionals [11,12,13] and large basis sets or even wavefunction calculations [14,15] for smaller molecules are then feasible. Such models are sensible when supporting experiments done in solution (e.g., cyclic voltammetry, UV–VIS (ultraviolet and visible) spectroscopy), where implicit solvation models [16,17] can work well. However, such models are incapable of capturing phenomena which are intrinsically inter-molecular, such as charge transport, formation of large excitons, or intermolecular charge transfer bands in optical spectra. Studies of mechanical properties [18] and defects [19,20] of organic solids also require explicit consideration of the aggregate state but are not the subject of this paper. Specifically, charge transfer calculations and ionic insertion and transport (which are critical phenomena in organic batteries) require going beyond single molecules and including aggregate-state effects explicitly. Another interesting phenomenon often observed in experimental literature is the effect on electronic properties (such as the oxidation potential or visible absorption peak) of alkyl chain length [21,22,23]. This effect is all but impossible to capture at the single-molecule level. Frontier orbitals which determine these properties are typically localized on the conjugated cores and are not sensitive to chain length. Interactions with neighboring molecules, however, can induce stress or conformations which can affect even electronic properties which are due to individual molecules (such as intramolecular excitations or monomer frontier orbital energies). Naturally, alkyl chains affect packing and with it the charge transfer integral. 

Modeling of aggregate-state effects is onerous, as packing may not be known and a larger system needs to be modeled. Specifically, for amorphous materials, such as the amorphous or semi-amorphous polymers often used in devices, large simulation cells are required. This poses difficulties for DFT modeling due to the near-cubic scaling of Kohn–Sham DFT. The use of hybrid functionals (which can provide quantitative gaps etc.) often becomes impractical, especially in periodic simulations of organic solids. Even with GGA (generalized gradient approximation) functionals, the CPU cost is substantial, requiring recourse to smaller basis sets and pseudopotentials. When computing optical properties with TD-DFT (time-dependent DFT), there is an additional inconvenience in that one needs to significantly increase the number of included states (to hundreds in the models described below). For these reasons, the use of semi-empirical methods such as density functional tight binding (DFTB) [24,25] and time-dependent DFTB (TD-DFTB) [26,27] is promising for systems wherein there are reliable DFTB parameterizations. Indeed, we previously showed that DFTB may have not just a CPU cost but also an accuracy advantage over GGA DFT for large systems [28]. 

Besides the issues of computational cost of (TD)-DFT with larger model sizes needed when modeling molecular solids and aggregates, there are issues beyond CPU cost which are specific to molecular aggregates and solids. Perhaps the most prominent is treatment of dispersion interactions which is deficient in common DFT functionals. While vdW (van der Waals) functionals are steadily making headway [29], the most widely used approach is a posteriori corrections with Grimme schemes [30,31,32] which can provide good structural models of and interaction energies in vdW systems. Grimme corrections are applicable to both DFT and DFTB. While for purely organic materials, these schemes perform well when (as is typically done) the corrections are applied to all types of atom, we will highlight below cases wherein selective (by atom type) application is preferred. For optical properties, due to the high CPU cost of TD-DFT, the dipole approximation [33,34], widely used for modeling of inorganic solids, has found use in the modeling of organic materials as well. Below, we will highlight works comparing its performance to TD-DFT(B) and to an alternative method developed by us. Obviously, for charge transport (electronic or ionic), explicit consideration of packing is critical, but for other properties, a practically important question is: to what extent is single-molecule modeling (enticing by its simplicity and speed) useful, even if the material is ultimately used in solid state? 

In this perspective, we summarize recent studies in the author’s group of aggregation effects on electronic properties of organic materials used in optoelectronic devices and in organic batteries [35,36,37,38,39,40,41,42,43,44,45,46,47,48,49,50]. This is not a review of materials or devices or of modeling methods and results, whose descriptions are already abundant in the literature, but a perspective based on our experience of specific issues in the modeling of these types of materials which are due to molecular aggregation. Specifically, we were able to compare optoelectronic properties computed with single-molecule and aggregate (clusters or solids) models and with different methods.

We show that in some cases it is possible to understand the mechanism and predict specific performance characteristics based on simple molecular models, while in other cases the inclusion of effects of aggregation is essential. For example, it is possible to understand the mechanism and predict the overall shape of the voltage-capacity curve for insertion-type organic battery materials, but not the absolute voltage. On the other hand, oligomeric models of *p*-type organic electrode materials can allow for relatively reliable estimates of voltages. Inclusion of aggregate state modeling is critically important for estimating charge transport rates, the effects of alkyl chain length, or when intermolecular charge transfer bands are important. In these applications, we highlight the use of alternative to (TD-)DFT approaches, such as (TD-)DFTB which significantly cuts the CPU cost of the modeling, and an alternative approach we proposed for computing absorption spectra which may be advantageous for aggregates [43,44].

## 2. Effect of Aggregation When Modeling Optoelectronic Properties

### 2.1. Absorption Spectra and Band Alignment

When molecules aggregate, their frontier orbitals undergo splitting and eventually band formation. They may or may not become delocalized over multiple molecular units. The effect on band alignment of aggregation comes from the difference in energy between the valence band maximum (conduction band minimum) and the HOMO (LUMO) of each molecule. The effect on the absorption spectrum comes on one hand from the modification of intramolecular transition intensities and energies due to perturbation exerted by neighboring molecules, even if the transitions are between molecular states that preserve their identity in the aggregate, and on the other hand from the possibility of inter-molecular charge transfer transitions and transitions between aggregate-specific states (bands) not existing in individual molecules. Intermolecular transitions especially, as charge transfer transitions in general, pose problems for TD-DFT modeling that are well-documented elsewhere [51]. These difficulties are among other things related to the high sensitivity of the governing TD-DFT equations [26,27,52] to orbital shapes and energies. This comes from the use of the integrals

(1) $$K_{ia\mu ,jb\nu }=\iint \phi_{i\mu }^{*}\left(r\right)\phi_{a\mu }\left(r\right)\left(\frac{1}{\left|r-r^{\prime }\right|}+\frac{\delta^{2}E_{XC}}{\delta \rho_{\mu }\left(r\right)\delta \rho_{\nu }\left(r^{\prime }\right)}\right)\phi_{j\nu }\left(r^{\prime }\right)\phi_{b\nu }^{*}\left(r^{\prime }\right)drdr^{\prime }$$

where indices *i*, *j*, and *a*, *b* label occupied and virtual orbitals *ϕ*, respectively; indices *μ* and *ν* denote spin; *ρ* is the density; and *E_XC_* is the exchange-correlation energy [52]. For example, the effect of errors in orbitals is much stronger on TD-DFT excitation energies than it is on orbital energies [53]. These issues carry into TD-DFTB.

Besides these in-principle difficulties with TD-DFT, there are difficulties in modeling spectra in the solid state when using periodic calculations, as hybrid functionals become very CPU-costly and TD-DFT is only implemented at the Γ point (i.e., without Brillouin zone integration) in popular periodic codes. In solid state, the so-called dipole approximation is popular [33,34], which is easily amenable to Brillouin zone integration and is often used in conjunction with GGA functionals which still dominate periodic calculations. In it, the imaginary part if the complex dielectric function $\epsilon_{i}\left(\omega \right)$ is computed:

(2) $$\epsilon_{i}\left(\omega \right)=\frac{2e^{2}\pi }{\Omega \epsilon_{0}}\underset{k,\nu ,c}{\sum }{\left|\langle \psi_{k}^{c}\left|q\cdot r\right|\psi_{k}^{\nu }\rangle \right|}^{2}\delta \left(E_{k}^{c}-E_{k}^{\nu }-\hbar \omega \right)$$

where Ω is the simulation cell volume; indices *ν* and *c* scan occupied and unoccupied $\psi_{k}^{c,\nu }$ orbitals (whose eigenstates are $E_{k}^{c,\nu }$), respectively; ***k*** is the wavevector; and ***q*** is the photon polarization vector. The real part $\epsilon_{r}\left(\omega \right)$ of the dielectric function is then computed from the Kramers–Kronig relation and then the absorption spectrum (absorption coefficient):

(3) $$\epsilon_{r}\left(\omega \right)=\frac{\sqrt{2}\omega }{c}{\left(\sqrt{\epsilon_{r}{\left(\omega \right)}^{2}+\epsilon_{i}{\left(\omega \right)}^{2}}-\epsilon_{r}\left(\omega \right)\right)}^{\frac{1}{2}}$$

Molar absorptivity can be obtained by multiplying σ(ω) by molar concentration. 

We recently introduced an alternative approach [43,44], in which one first computes the real part of the complex dielectric function from the ab initio-computed frequency-dependent polarizability α(ω) using the Clausius–Mossotti relation [54]

(4) $$\frac{\epsilon_{r}\left(\omega \right)-1}{\epsilon_{r}\left(\omega \right)+2}=\frac{N\alpha \left(\omega \right)}{3\epsilon_{0}}$$

where *N* is the numbers density of molecules and $\epsilon_{0}$ the permittivity of vacuum. The Clausius–Mossotti relation makes the so-called Lorentz local field approximation that the long-range interactions are isotropic and that there is no charge transfer between molecules, which is a reasonable approximation for some organic materials; for example, C60. A conceptual advantage of this method is that the polarizability could in principle be computed without orbitals (although in popular codes such as Gaussian, the polarizability is computed from orbitals). A practical advantage is that of parallelizability, as $\epsilon_{r}\left(\omega \right)$ can be computed for each frequency separately.

**Figure 1:**
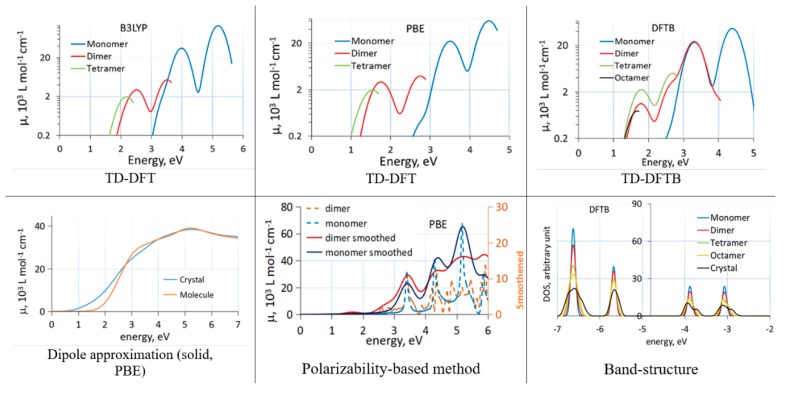
Absorption spectra of C60 clusters (TD-DFT(B) and polarizability-based method) and solid (dipole approximation) computed with different methods. TD-DFT(B) curves end due to a finite number of excited states. The bottom right panel shows changes to the bandstructure induced by aggregation. Reproduced from [43] with the permission of AIP Publishing.

Comparisons between different methods when computing effects of molecular aggregation are rare. We recently studied the effect of aggregation on band alignment and optical absorption of C60 [43]. We used cluster models of C60 aggregates (cut out of the known face-centered cubic structure of C60 [55]) and compared the effect of aggregation on optical absorption with the three methods. Figure 1 shows absorption spectra of C60 clusters and the solid with different methods. We observed that the dipole approximation and TD-DFT with a GGA functional (PBE) induce a large artificial redshift. In clusters, this could be related to strongly artificially redshifted intermolecular charge transfer transitions. Interestingly, the polarizability-based approach was able to give a reasonable spectrum of clusters even with the GGA functional, in line with experimental comparisons between C60 spectra in solid and in solution [56,57]. 

The (dispersion corrected) DFTB method, in spite of showing an artificially strong redshift due to aggregation and an overall redshift due to parameterization based on GGA calculations, is very convenient for comparative studies between molecules, clusters, and solids, as its cost advantage allows for routine calculations on large clusters and solids of organic materials. In TD-DFTB, hundreds of excited states can easily be considered, and band structure changes between molecules, clusters, and solids can easily be computed. In Figure 1, the band structure changes due to aggregation of C60 molecules computed with DFTB are also shown. 

The computational cost advantage of DFTB allows for routine calculations of solid organic materials, including structure optimization and molecular dynamics (MD) simulations to find structures. Molecular packing information obtained with DFTB can be used for charge transport rate calculations with other methods (see below). In [39], we compared multiple fullerene derivatives differing by the core (C60 or C70) and type and number of addends (mono- or bi-addend). These are actively researched as acceptor and electron transport materials in OSC and PSC. While for some of the derivatives experimental structures had been determined from XRD (X-ray diffraction) measurements and used in the model, for others, we used DFTB MD to find plausible structures. We found that the crystal packing leads to changes of LUMO and HOMO of about 0.2 and 0.1 eV, respectively, and hence, a change in band alignment (with e.g., a donor) and in the driving force to charge transfer which is expected to have a noticeable effect on charge separation (due to exponential sensitivity of the charge transfer rate to driving force; see below). 

An often neglected phenomenon in the modeling of materials for optoelectronic devices are nuclei vibrations, which can strongly affect band alignment and charge transfer rates. In [48] we modeled the effect on band structure of solid C60 and C70 (with known face-centered cubic and hexagonal closed packed structures [55,58]) by performing DFTB MD. As shown in Table 1, nuclei motions can modify the HOMO and LUMO values by amounts comparable to reorganization energies and driving forces, which is expected to affect significantly charge separation.

**Figure 2:**
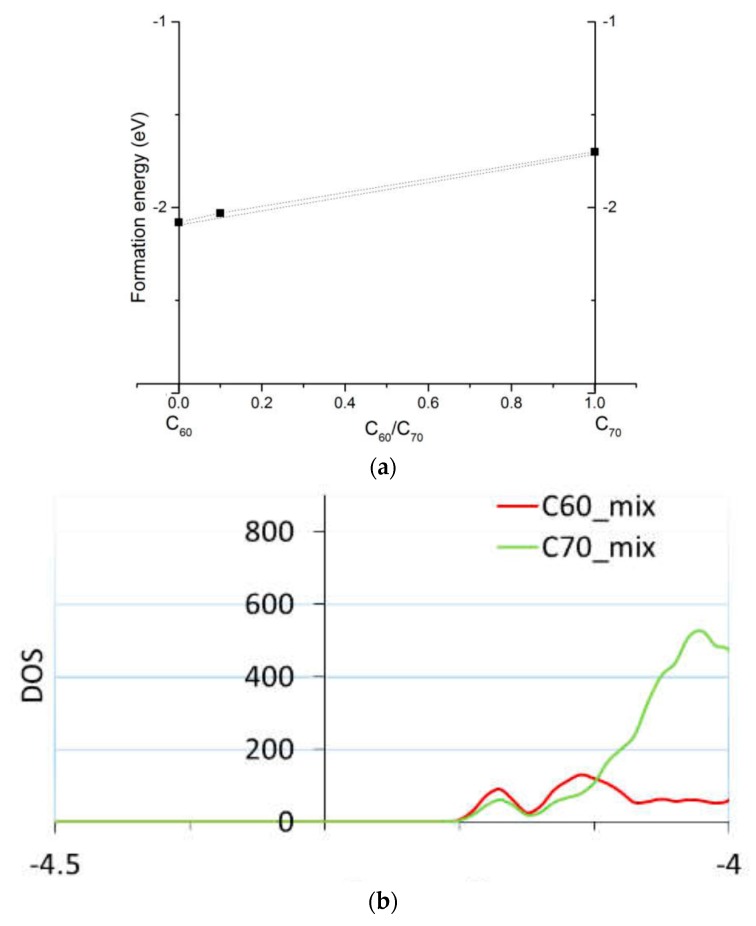
The computed formation energies (**a**) of C60, C70, and the mixture of C70 and C60; and a zoom in into the density of states (DOS) of the mixture (**b**) showing separate C60 and C70 contributions. Adopted with permission from the Supporting Information of [42]. Copyright (2018) American Chemical Society.

While simulations of homo-molecular organic solids can be done with small simulation cells (as small as the unit cell), mixed materials intrinsically require larger cells and are correspondingly costlier to simulate. For example, in [42] we considered the effect on electronic properties of a mixture of C60 and C70. Such a mixture with about 1/10 fraction of C70 was shown experimentally to improve the performance of planar perovskite solar cells compared to pure C60 or C70 electron transport layers [42]. We performed DFTB calculations of solid solutions of C60 and C70 (based on known crystal structures of these fullerenes [55,58]), both in bulk and on surfaces, to verify whether segregation would occur into separate C60 or C70 domains and whether there is an effect on charge transport. We computed that there is minimal driving force to segregation (on the order of room-temperature *k_B_T*, where *k_B_* is the Boltzmann constant and *T* the temperature) and negligible effect on band alignment; see Figure 2.

**Figure 3:**
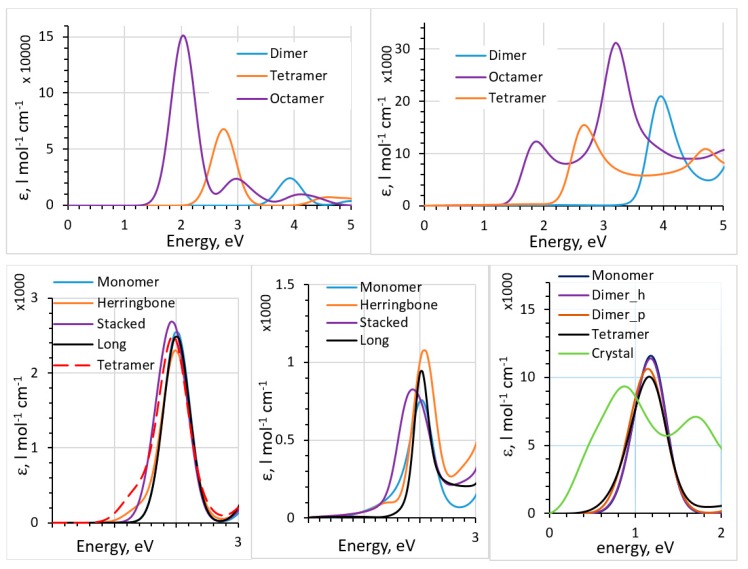
Top: absorption spectra of thiophene oligomers computed with TD-DFT (top left) and the polarizability-based method (top right), with B3LYP functional. Bottom: absorption spectra of different pentacene clusters (see [44] for definitions of the clusters) cut out of the crystal structure computed with TD-DFT (bottom left) and the polarizability-based method (bottom middle), with B3LYP functional. Bottom right: absorption spectra of solid pentacene and different pentacene clusters cut out of the crystal structure computed with the dipole approximation, with PBE functional. See [44] for details. Reproduced from Ref. 44, with the permission of AIP Publishing.

The particularity of fullerenes is that, contrary to most organic dyes and often-used polymeric donors, the absorption peak is not dominated by the HOMO-to-LUMO transition but is composed of a large number of transitions involving many orbitals. We also compared TD-DFT, the dipole approximation, and the polarizability-based method for such systems [44]. In Figure 3, we show the results of comparison between the TD-DFT and the polarizability-based method for thiophene oligomers and pentacene clusters (based on the known crystal structure of pentacene [59]). For pentacene, a comparison of the spectra of clusters and of the solid pentacene computed with the dipole approximation is also given. Dyes were also considered in [44]. We observed that the polarizability-based method provided excitation energies similar to those with TD-DFT (and therefore similar accuracy with respect to experimental data [53,60,61,62]) but can have very different (lower) intensities. Contrary to TD-DFT, which produces stick spectra which are then artificially broadened, the polarizability-based method results in continuous spectra (which typically still need to be broadened [43,44]). Intensities are also affected to a larger degree than peak positions by the approximations inherent in the method (such as the convergence of the Kramers–Kronig integral and the accuracy of the Clausius–Mosotti relation). We observed strong and unrealistic redshift with the dipole approximation when going from small clusters to a solid (last panel of Figure 3). This signals that further method development should be pursued for practical yet accurate methods to compute spectra of solids beyond the dipole approximation and TD-DFT.

We also recently computed the effects of molecular aggregation on the optical properties of naphthalene flanked diketopyrrolopyrroles (DPPN) with different alkyl chains; specifically, hectyl (H) and octyl (O). As described in the Introduction, alkyl chain lengths represent an interesting degree of freedom in molecular design, but their effect on electronic properties is largely missed at the single-molecule level. We computed [50] and compared absorption spectra of molecules with those of clusters of about a dozen molecules and with those of solids, modeled based on XRD data. Dispersion-corrected (TD-)DFTB was used to account for the relatively large size of the systems, which, in particular, required the inclusion of hundreds of excited states to capture the peak of visible absorption. TD-DFTB results were benchmarked to TD-DFT with a hybrid functional for single molecules: while (TD)-DFTB resulted in underestimated band gaps and excitation energies (as is expected given that the DFTB parameterization is based on GGA calculations), it can be used for qualitative assessment and is reliable for comparison between the two molecules and for evaluation of the effects of packing. We observed that at the single-molecule level, the electronic and optical properties are not affected by the difference in chain length, as expected. Aggregation, however, induced changes in HOMO, LUMO, and the gap on the order of tenths of an eV vs. single molecules, and these changes were different on the order of 0.1 eV for different alkyl chains. Specifically, the cluster calculations (which compute orbital energies with respect to the vacuum level, contrary to periodic calculations) resulted in higher HOMO values vs. single molecules, by 0.2–0.3 eV. Both cluster and periodic calculations showed a decrease in the gap in H-DPPN vs. O-DPPN due to aggregate-state effects, by about 0.3 eV. The optical adsorption peak of H-DPPN was computed to be slightly red-shifted (by 25 nm) vs. that of O-DPPN. The modeling was in agreement with the experiment [50]. Computations of effects due to alkyl chain size, which are well known in experimental literature, require, therefore, explicit consideration of molecular packing.

### 2.2. Charge Transport

To evaluate the electron or hole transport capability of organic materials, often, the Marcus theory is used, as opposed to band transport suitable in solid inorganic semiconductors. The Marcus theory essentially assumes a hopping mechanism which is usually associated with small polarons, which may or may not be the case in organic materials. In Marcus theory, the rate is

(5) $$\omega_{ij}=\frac{{\left|J_{ij}\right|}^{2}}{\hbar }\sqrt{\frac{\pi }{\lambda k_{B}T}}\exp\left[-\frac{{\left(\Delta G_{ij}+\lambda \right)}^{2}}{4\lambda k_{B}Τ}\right]$$

where $\omega_{ij}$ is the charge transfer rate between states *i* and *j*, *λ* is the reorganization energy, and $\Delta G_{ij}$ is the driving force (difference in Gibbs free energies between the two states). *J_ij_* is the overlap integral between the wavefunctions of states *i* and *j*: $\left\langle \psi_{i}|V_{ij}|\psi_{j}\right\rangle$, where *V_ij_* is the coupling (Coulomb interaction) term. The driving force could be approximated by the differences between corresponding single electron state energies. The Marcus equation depends strongly and non-linearly on the driving force and on intermolecular separation. The overlap integral *J_ij_* strongly depends on mutual position of molecules and its proper estimate requires explicit aggregate state modeling. *J_ij_* can in certain cases be estimated from the orbital splitting (e.g., for nearly-isotropic interactions and when molecular sites are similar) [63], but in general, more involved methods need to be used, such as dimer projection [64,65] which was also used by us in our studies of charge transport in fullerene derivatives. The dimer projection method has an advantage over the orbital splitting approach in that it does not make the isotropic approximation [63]. In the dimer projection method, the charge transport integral is computed as $\left\langle \psi_{i}|F_{ij}|\psi_{j}\right\rangle$ where the states $\psi_{i}$, $\psi_{j}$ are represented by frontier orbital energies of two isolated molecules in the dimer, and $F_{ij}$ are elements of the Fock matrix $F=SC\epsilon_{KS}C^{-1}$ computed from the overlap matrix ***S***, the matrix of orbital coefficients ***C***, and the vector of Kohn–Sham energies $\epsilon_{KS}$ [63,64,65].

In [39] we computed electron and hole transport rates of multiple fullerene derivatives (different mono and bi-adducts on C60 and C70) based on DFTB-simulated solid state structures, using the Marcus theory and the dimer projection method. Reorganization energies were estimated from single-molecule calculations. The electron and hole transfer rate computed with this approach could vary by orders of magnitude depending on the addends. The reference value for solid C60 computed with Marcus theory (5.87 × 10^12^ s^−1^) was in good agreement with available literature [66,67]

In [42] we also computed the effect on electron transport of a relatively small amount (on the order of 10 mol%) of C70 in C60, which, as mentioned above, was shown to improve the performance of PSCs. Solid state structures were computed with DFTB, and key dimers cut out of the solid structure were used to compute electron transport rates. The computed electron transfer rates that can be achieved with C60/C70 mixed structure, based on rates between C60/C70 units, were in the order of 10^12^ s^−1^ (highest 3.98 × 10^12^ s^−1^). The important conclusion was that the electron transfer rate does not noticeably drop compared to pure C60 (on the order of 5 × 10^12^ s^−1^) crystals (for comparison, the rate was 1.8 × 10^13^ s^−1^ for pure C70).

**Figure 4:**
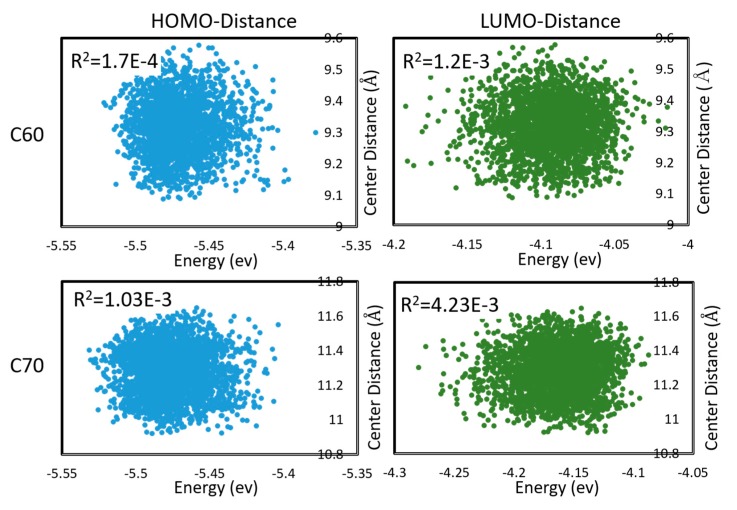
Correlations between HOMO and LUMO energies, and intermolecular distances (between centers of mass) during molecular dynamics in C60 and C70. Pearson *R^2^* coefficients are also shown. First appeared in [48].

To properly estimate charge transfer rates under a realistic distribution of mutual geometries of monomers, and under thermal motions, one is required to sample the configuration space of both inter- and intra-molecular degrees of freedom and take a statistical average. This could be very costly. In [48], we considered the electron and hole transfer rates in C60 and C70 (understood as being due to hopping between LUMO and HOMO, respectively, of neighboring molecules) averaged over DFTB molecular dynamics. C60 and C70 units were found to be mobile during MD; i.e., the trajectories sampled both intramolecular and intermolecular (mutual positions of molecules) degrees of freedom. We found that effects on the Marcus equation via the changes in the driving force (via changes in HOMO, LUMO energies) and via the changes in the integral are largely uncorrelated, as shown in Figure 4. The absence of correlation permits estimating the expected charge transfer rate with the effect of nuclear motions and with respect to the distribution of inter-molecular geometries $\left\langle \omega_{ij}\right\rangle$ as

(6) $$\langle \omega_{ij}\rangle =\frac{\left\langle {\left|J_{ij}\right|}^{2}\right\rangle }{\hbar }\sqrt{\frac{\pi }{\lambda k_{B}T}}\langle \exp\left[-\frac{{\left(\Delta G_{ij}+\lambda \right)}^{2}}{4\lambda k_{B}Τ}]\right\rangle$$

where the angular brackets denote expectation values (averages over MD trajectories). Due to the strongly non-linear dependence, the averages are different from the integrals, Marcus exponents, and rates computed at the equilibrium geometry several-fold. This uncoupled approximation significantly simplifies calculations.

## 3. Effect of Aggregate State When Modeling Organic Battery Materials

### 3.1. Insertion-Type Materials

Insertion-type organic electrode materials, often called *n*-type materials, operate on the same principle as traditional inorganic materials; i.e., Li, Na, or Mg (etc.) cations reversibly insert into the electrode material where they coordinate to reduced host molecules. The reduction potential or LUMO of the material is therefore a critical determinant of the voltage, and LUMO design, by the choice of key building blocks and functional groups, is an important component of the material’s design. Many types of molecular materials have been studied as *n*-type electrode materials, including quinones, carboxylates, tetracyanides, etc. [68] Such materials are typically used as anodes; very deep LUMO levels would be required [69] to achieve voltages in excess of 3 V, which is possible, e.g., with cyanides [37,70].

Computational support for studies of insertion-type materials included both solid state simulations and molecular simulations [49]. In solid state modeling [35,37,40,71], the methods which have been established for inorganic materials are then applicable. Specifically, voltages are computed from insertion energetics [72]:

(7) $$V=-\frac{E\left(M_{y+m}Host\right)-E\left(M_{y}Host\right)-mE\left(M\right)}{nmF}$$

where *V* is the voltage between *M* (*M* = Li, Na, …) concentrations corresponding to *y* and *y* + *m* atoms of *M*, *E*(*M_y,y+m_Host*) are energies of the host in the corresponding charge states and *E*(*M*) the energy per atom of bulk *M*, *n* is the number of electrons transferred per unit *M* (*n* = 1 for Li or Na, *n* = 2 for Mg etc.), and *F* is the Faraday constant.

Molecular simulations are widely used [36,38,41,73,74,75] and attractive by their simplicity and overwhelming CPU cost advantage. Studies which compare the results of molecular and solid state modeling are, however, scarce. Such studies are important to understand what can be gained from simple molecular models and what is irretrievably lost in them. We produced such studies for several organic materials, including tetracyanoethylene (TCNE) [37,38], disodium terephthalate [35,36], and sodium pyridine dicarboxylate [40,41]. For these materials, we have used available XRD-based structures for solid state modeling and achieved with those models good matches with measured voltages [35,37,40] (see these references for detailed comparisons to the experiment); these models, together with experimental data, can in turn serve as benchmarks for molecular models [36,38,41,47]. In molecular models, cation attachment energies are also used to estimate voltages [36,41,73]; an alternative approach which is used to estimate the voltage with molecular materials is estimation of the reduction (or oxidation for *p*-type materials, see below) potential of the molecule, which in the simplest case can be computed from the energies of the neutral and oxidized or reduced molecule [76]:

(8) $$E_{ox/red}=-\frac{1}{n}\left(E_{neutral}-E^{n+/-}\right)$$

where *n* is the number of electrons involved in the oxidation reaction. The redox potential, when related to the M/M^+^ potential, gives an estimate of the voltage in a specific type of battery.

**Figure 5:**
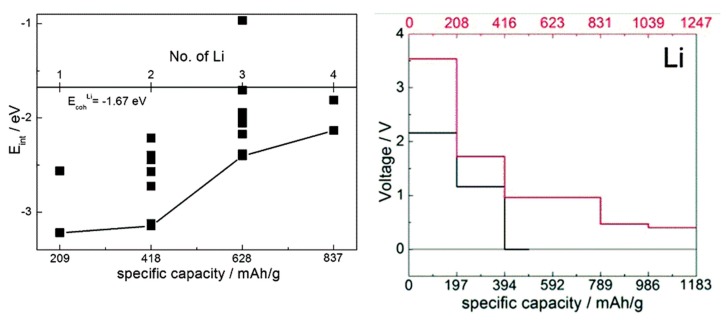
Left: computed interaction energy of Li with a TCNE molecule, with a hybrid functional. Different symbols correspond to different configurations. The distance from the black curve to the cohesive energy of Li gives an estimate of voltage. Adapted from [38] with permission from the PCCP Owner Societies. Right: computed voltage-capacity curves for lithiation of solid TCNE (red curve and axis) and LiTCNE MOF (black curve and axis), using a GGA functional. Adapted from [37] published by the PCCP Owner Societies.

When comparing estimates of voltage-capacity curves obtained from interaction energies with the cations obtained with single-molecule calculations and with solids [35,36,37,38,40,41], we observed that on one hand, mechanistic insight can be obtained already from molecular calculations (showing, for example, LUMO occupancy by Li valence electrons [37,38] or preferred coordination to redox active groups [40,41]) and the overall shape of the voltage-capacity curve up to a state of charge typically corresponding to full LUMO occupancy (e.g., two cations per molecule) [35,36,40,41,47]. Molecular calculations can serve well to interpret IR spectra used in the assignment of the mechanism of charge–discharge [47]. The absolute voltages are, however, underestimated on the order of an eV due to neglect of the environment. This is illustrated in Figure 5 on the example of TCNE (for comparison, the open circuit voltage computed for sodiation of the vdW TCNE crystal, at 3.3 V [37], is in excellent agreement with the experimental value of 3.3 V [77] which was not known to us at the time of modeling). This example also illustrates that for the same active molecule, the solid state environment can be very different, resulting in a very different voltage-capacity profile: in the case of TCNE, the host can be a vdW crystal or a covalently-bound LiTCNE MOF (metal-organic framework) [37].

Because molecular calculations do not take into account the steric environment, they tend to predict more protracted voltage-capacity curves than are observed experimentally; i.e., they do not naturally indicate final state of charge. We recently showed, however, that the final state of charge can be deduced in molecular calculations from the changes in the degree of ionization of Li [74]. Artificial parts of voltage-capacity curves from molecular calculations are often due to post-LUMO occupancy. While this may be an artefact in some materials, this also suggests that realizing post-LUMO occupancy is an interesting design strategy of high-capacity materials; recent experimental results seem to confirm this [78].

A significant advantage of molecular models is ease of use of hybrid functionals which are generally more accurate. This is in contrast to solid state modelling, which is still dominated by GGA functionals. In [74] we showed, by comparing hybrid and GGA calculations of lithiation of C60, that mechanistic details could be missed in GGA calculations (such as changes in energies of key electronic states). On the other hand, encouragingly, voltage-capacity curves were similar (differing on the order of only 0.1 V) between the two types of calculations, supporting the continued use of GGA functionals for cases where hybrids are too costly.

**Figure 6:**
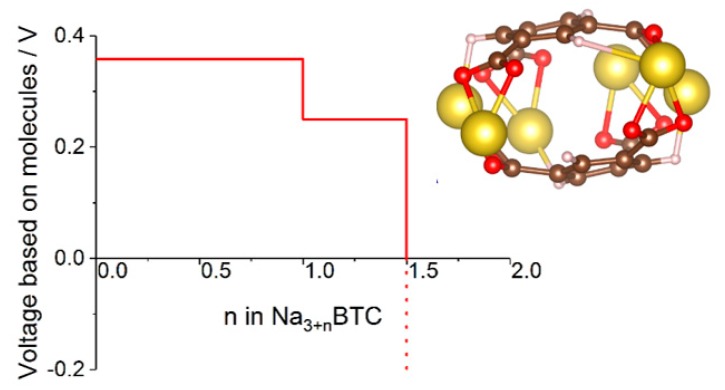
Voltage-capacity curve of sodium benzene *tri*carboxylate computed with a cluster model shown in the insert. See [47] for details.

In some cases where molecular calculations cannot provide a good model even qualitatively because environmental effects are critically important for the mechanism, one can resort to cluster models. This was done in [47], where a cluster model of sodium benzene *tri*carboxylate was used to compute the voltage-capacity curve (Figure 6) which reproduced well the experimental curve and IR spectral features used to establish the mechanism. The model helped establish a new mechanism whereby reversibly inserted Na atoms are not fully ionized, contrary to other known dicarboxylate materials [47].

For solid organic materials, there is tendency to use dispersion-corrected DFT, often with Grimme-type corrections. Indeed, this is necessary to obtain correct structures of vdW crystals. We have shown, however, that dispersion corrections may not be necessary in MOF-type materials like Li or Na carboxylates and that they may significantly worsen voltage estimates [35] (see the data in Supporting Information in [35] which include comparison with the experiment). Even in vdW crystals, we found that it is best to exclude the corrections for atom pairs including fully ionized Li or Na [37]. The rationale for this is that once the valence shell has been fully ionized, there is no basis for the correction.

The DFTB method appears enticing to model solid organic electrode materials. It would not only speed up the calculations of voltage-capacity curves but could enable direct “ab initio” studies of the dynamics of charge discharge. In our studies of these insertion-type materials, we found, however, that existing parameterizations could not provide quantitative (insertion energy or voltage) or qualitative (energetic ordering of different insertion sites) accuracy. This is not an in-principle DFTB limitation; parameterizations involving Li and Na which are specific to organic battery materials are needed.

### 3.2. p-Type Materials

In *p*-type organic active electrode materials, the active metal cation (such as Li^+^, Na^+^) does not insert into the cathode material during battery discharge. Instead, the metal cations reversibly coordinate to the anions of the salt in the electrolyte, such as ClO_4_^−^ or PF_6_^−^. The cathode material is reversibly oxidized by coordination and de-coordination to the anions [6]. The voltage is largely determined by the oxidation potential or the HOMO, and HOMO design, through the choice of key building blocks and functional groups, is an important component of the material’s design. This type of material is naturally more suited to realize high voltage (and therefore high energy density) organic cathodes. Most proposed *p*-type materials are polymers. Even common and non-expensive polymers such as polythiophene or polyaniline (PANI) can be used as cathode materials. This practically important and promising class of materials poses difficulties in ab initio modeling. Modeling of solid polymers requires relatively large simulation cells; moreover, these materials are often amorphous or semi-amorphous, which requires large simulation cells (on the order of 10^3^ atoms or more) and consideration of multiple conformations.

This is where the CPU cost advantage of the DFTB method is very promising. The difficulties with current parameterizations involving metal atoms can be obviated, as a model of the *p*-type cathode need not include Li or Na, and existing DFTB parameterizations are sufficiently accurate to model polymer-counteranion combinations. This is the case specifically for polyaniline interaction with ClO_4_^−^. In [46], we estimated the voltage curve of PANI based on single-molecule DFT calculations of the oxidation potential of PANI using small oligomers. In [45], we computed the voltage-capacity curve of solid amorphous PANI in a study that combined force field MD (to pre-optimize and sort multiple structures by energy), DFTB (to optimize a pre-selected number of structures), and DFT (to benchmark DFTB). As each simulation cells contained on the order of 10^3^ atoms and dozens of structures had to be considered at different degrees of oxidation of PANI (different concentrations of counter-anions), the use of DFTB was critical for feasibility. The report in [45] presented the first relatively large-scale ab initio model of the voltage-capacity curve of a solid amorphous polymeric cathode.

**Figure 7:**
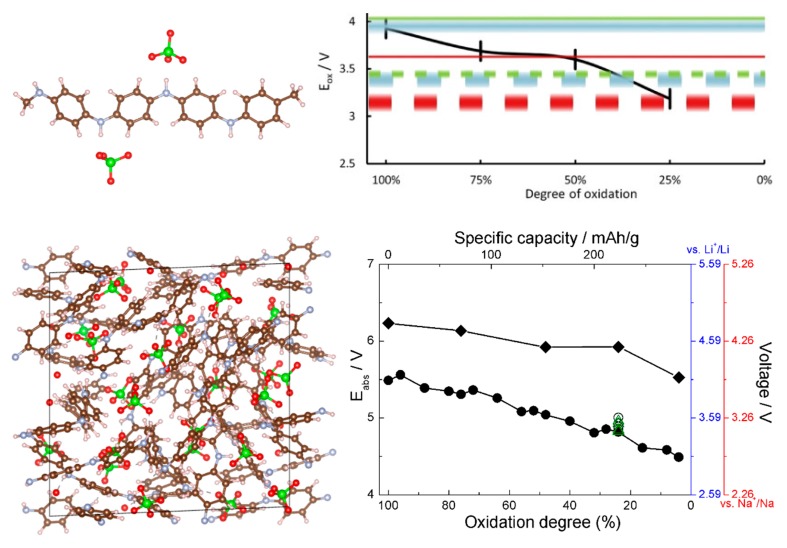
Top left: the structure of the oligomeric model of PANI showing coordination of ClO_4_^−^ anions. Top right, black curve: the computed voltage profile (for a Li ion battery) from the oligomeric model. Reproduced from [46] with permission from Elsevier. Bottom left: a simulation cell of solid PANI with intercalated ClO_4_^−^. Bottom right: the computed voltage profile for Li and Na ion batteries from the solid state model for PANI (curve with round symbols) and CN-functionalized PANI (curve with rhombic symbols). Reproduced from [45] with permission from the PCCP Owner Societies.

References [45,46] thus allow comparing voltage-capacity curves computed from small oligomers and from solid state calculations. This is shown in Figure 7; the agreement between the solid state and molecular models is quite good, which bodes well for usability of molecular models for modeling of *p*-type materials. The agreement with the experimental data, also given in Figure 7, is also good. This is practically important as it much simplifies computational screening of new materials via selection of aromatic cores, functionalizations, and dopants [79,80]. This was done in [46], where a number of functional groups were tested on an oligomeric model and functionalization with cyano groups was identified as achieving simultaneously higher chemical stability and higher voltage. Voltage curves for CN-functionalized solid PANI were also computed in [45], and they agreed well between oligomer and solid state calculations [45,46].

## 4. Conclusions

We considered the effects of molecular aggregation on optoelectronic properties important for solar cells and LEDs (such as absorption spectra and electron and hole transport rates) and properties important for use of organic materials as active electrode materials in metal ion batteries (redox potentials and cation storage properties).

While absorption spectra and redox potentials can be computed at the single-molecule level, they are modified by molecular aggregation. For calculations of rates, specifically within the Marcus theory, explicit consideration of intermolecular arrangements is necessary. The DFTB approach provides a useful way to compute structures of organic solids (which often require large simulation cells costly with DFT), even if electronic properties are computed with higher-level methods. It is also useful for comparative studies; e.g., to capture the effect of aggregation on band structure/alignment, or with TD-DFTB, on spectra.

The staple methods for computing spectra, TD-DFT (and TD-DFTB) widely used in molecular modeling, and the dipole approximation widely used in solid state, often result in artificially redshifted bands due, in particular, to intermolecular transitions. We introduced an alternative approach based on frequency-dependent polarizability which in some cases can mitigate this problem. It deserves further studies, especially with regard to the possibility to avoid reference to orbitals.

It appears more and more important to include the effect on charge transport of distributions among molecules in an organic solid resulting from structural disorder and from thermal motions. One way to include said effect is by estimating expectation values of the Marcus rate. We showed that such estimates could be done in an uncoupled approximation whereby effects on the Marcus exponent (via Δ*G*) and on the overlap integral can be averaged separately, which is a practically useful simplification.

Just as in materials modeling for optoelectronic applications, in the modeling of materials for organic batteries, some properties are relatively accurately computable at the single-molecule level, while others need explicit consideration of the solid. Mechanistic details of charge–discharge and the shape of the voltage-capacity curve are often well reproduced with molecular models while the absolute voltages and final states of charge are not. When pre-screening materials, for example, when studying different functionalizations, however, relative changes in properties are sufficient to judge on the promise of a material and can be computed from molecular models. The final state of charge can sometimes be deduced from the analysis of charges even if the formally computed voltage curve with molecular models tends to drag beyond theoretical capacity.

We observed qualitative and quantitative agreement between voltage curves of *p*-type electrode materials computed with molecular and solid state models. This bodes well for the use of molecular models for computational screening of organic cathodes.

A key advantage of molecular models is ease of use of hybrid functionals, which in some cases is critical for correct assignment of the mechanism. When single-molecule calculations cannot provide a good model because environmental effects are critically important for the mechanism, a cluster model may be useful, preserving major advantages of molecular modeling (including use of hybrid functionals) while capturing some effects due to an aggregate state.

Similar to its advantage when modelling solid organic materials for optoelectronic devices, DFTB is advantageous for organic batteries as well. We found that work is still needed on accurate parameterizations for insertion-type materials which require inclusion of metal atoms in the parameter set. For *p*-type materials, however, existing parameterizations can be accurate enough, as metal atoms need not be included in the model.

## Figures and Tables

**Table 1:** HOMO and LUMO energies at the equilibrium geometry (“Equil.”), their expectation values over MD trajectories (“<…>”), standard deviations of HOMO and LUMO distributions over MD trajectories (“σ”), and reorganization energies λ and driving forces Δ*G*_eq_ for electron and hole transport in C60 and C70 computed in [48]. All values are in eV.

		Equil.	<…>	σ	λ	Δ*G*_eq_
LUMO	C60	−3.98	−4.09	0.023	0.132	0.15
	C70	−4.04	−4.16	0.027	0.123	0.15
HOMO	C60	−5.60	−5.47	0.019	0.167	0.23
	C70	−5.54	−5.48	0.020	0.138	0.34

## Abbreviations

CPU	central processing unit
DFT	density functional theory
DFTB	density functional tight binding
DOS	density of states
GGA	generalized gradient approximation
HOMO	highest occupied molecular orbital
LUMO	lowest unoccupied molecular orbital
MD	molecular dynamics
MOF	metal-organic framework
OLED	organic light-emitting diode
OSC	organic solar cell
PANI	polyaniline
PLED	perovskite light-emitting diode
PSC	perovskite solar cell
TCNE	tetracyanoethylene
TD	time-dependent
UV-VIS	ultraviolet and visible
vdW	van der Waals
XRD	X-ray diffraction
